# Deficits in Scaling of Gait Force and Cycle in Parkinsonian Gait Identified by Long-Term Monitoring of Acceleration with the Portable Gait Rhythmogram

**DOI:** 10.5402/2012/306816

**Published:** 2012-10-16

**Authors:** Hiroo Terashi, Hiroya Utsumi, Yohei Ishimura, Tomoko Takazawa, Yasuyuki Okuma, Mitsuru Yoneyama, Hiroshi Mitoma

**Affiliations:** ^1^Department of Neurology, Tokyo Medical University, Tokyo 160-0023, Japan; ^2^Department of Neurology, Shizuoka Hospital, Juntendo University, Shizuoka 410-2295, Japan; ^3^Mitsubishi Chemical Group Science and Technology Research Center, Inc., Kanagawa 27-0033, Japan; ^4^Department of Medical Education, Tokyo Medical University, Tokyo 160-0023, Japan

## Abstract

To examine the range of gait acceleration and cycle in daily walking of patients with Parkinson's disease (PD), we compared the gait of 40 patients with PD and 17 normal controls by using a newly developed long-term monitoring device that extracts gait-related accelerations from overall movements-related accelerations. The range of change in gait acceleration, relative to the control, was less than 75% in 12 patients. The range of change in gait cycle was less than 75% in 8 patients. The range of changes in both parameters was less than 75% in 4 patients. The results suggest narrow changes in gait parameters in PD.

## 1. Introduction

Parkinson's disease (PD) is a degenerative disease characterized by depletion of dopamine, which leads to the development of akinesia (poverty in movements generation) and bradykinesia (slowness in executed movements) [1–5]. These motor symptoms should be examined in daily life, since routine neurological examination in the hospital can detect only certain but not all aspects of motor abnormalities [2]. Long-term monitoring devices have been developed to analyze motor deficits that interfere with daily activities [6–10]. We also developed recently the portable gait rhythmogram (PGR, MGM-1100) that allows exact extraction of gait-related accelerations from overall motion-related accelerations during long-term monitoring [11, 12]. The “pattern matching method” is mathematically used to identify gait-related acceleration. Using the PGR, we have already described poverty of movements and deviation in gait rhythm, such as freezing of gait [11, 12], and addressed motor fluctuations by tracing these parameters [11].

Previous studies indicated that PD patients can only execute movements of different amplitudes at a single and slow velocity and cannot increase their movement velocity [13]. Another study reported limited peak EMG activity that can be generated in a muscle burst in PD patients [14]. To examine the hypothesis of “deficit in scaling” on gait, it is necessary to ask subjects to walk at various rhythms or powers and to quantify the changes in each gait parameter. Although this analysis is experimentally difficult, one potentially useful approach would be to examine data recorded during daily activities since in daily walking, subjects are obliged to vary gait strategies depending on the context, for example, easy walking or strenuous walking. Daily long walk could be a good sample for examining the range of changes in gait parameters. 

Using our newly developed PGR, we tested the hypothesis that PD patients find it difficult to shift gait parameters in response to varying situations during walking. In a previous study, we reported a limited range of gait acceleration in patients with severe akinesia [12]. Since gait acceleration reflects the floor reaction force, the results suggested that PD patients tend to walk with a narrow range of change in floor reaction force. The present study expands this analysis by comparing simultaneously the range of step cycle with that of acceleration. For this purpose, we measured the *gait acceleration-cycle plot *from 24 hr recordings. 

## 2. Methods

### 2.1. Subjects

Using our PGR, we recorded the daily walking profiles of 40 PD patients (26 men and 14 women) with a mean age of 66.2 ± 5.7 years (±SD) (Table 1). They represented all patients admitted to Tokyo Medical University Hospital between June 2009 and March 2010 who could walk unaided and showed no peak-dose dyskinesia during the “on” time. They included 8 patient with Hoehn and Yahr stage I, 9 with stage II, and 23 with stage III. The clinical status was examined using the Unified Parkinson's Disease Rating Scale (UPDRS) motor score “on” state (mean score of UPDRS Part III; 19.2 ± 7.1) [15]. The study also included 17 age- and height-matched normal controls (age, 64.7 ± 4.5 years, 8 men and 9 women). Matching for age and height was based on the finding that gait cycle and floor reaction forces are influenced by these two parameters.

Informed consent was obtained from all subjects. All procedures were conducted in accordance with the guidelines of the ethics committee of our institution. Statistical analysis was performed using the student's *t*-test. A *P* value of <0.05 denoted the presence of a significant difference.

### 2.2. Measurements

#### 2.2.1. Monitoring of Acceleration

**Figure 1:**
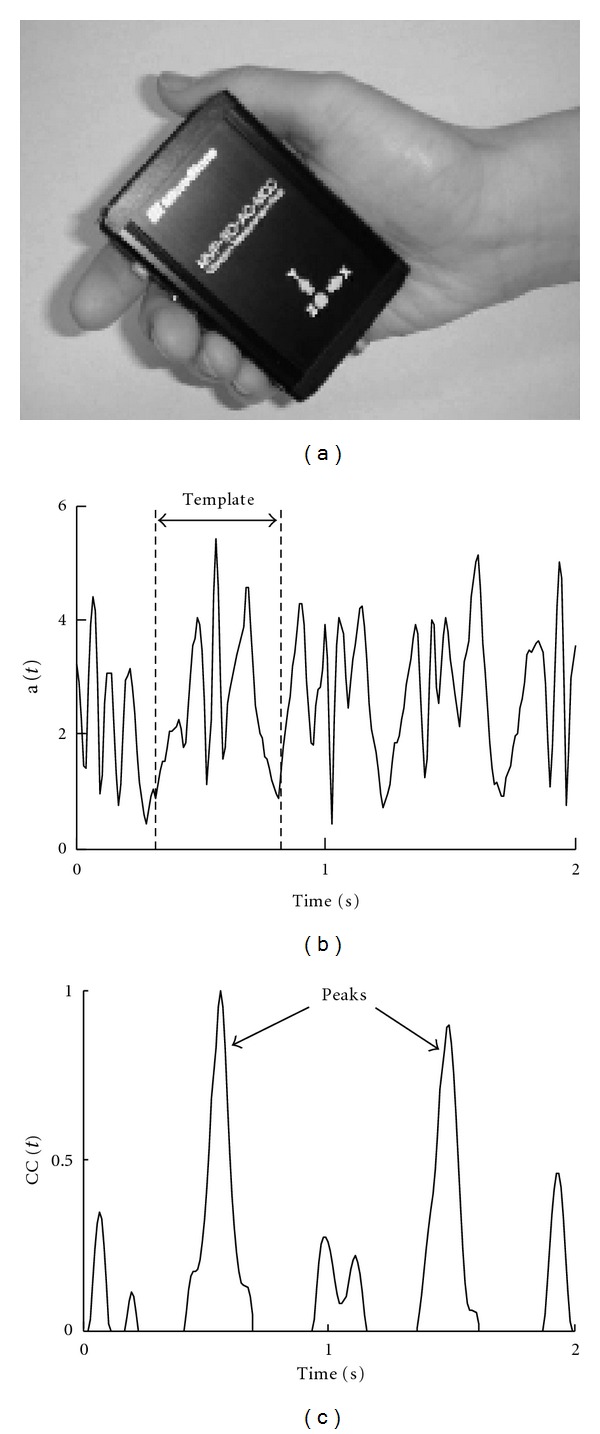
(a) The portable gait rhythmogram device. (b) Schematic presentation of the acceleration time series (a(*t*)), and a template for cross-correlation calculation. (c) Schematic presentation of the cross-correlation time series (CC(*t*)).

The portable PGR is a small device (size, 8 × 6 × 2 cm, weight, 80 g) (Figure 1(a)) that measures three dimensionally (*a*_*x*_, *a*_*y*_, *a*_*z*_) the accelerations accompanied by (1) limb and trunk movements and (2) those induced by step-in and kick-off during gait [11, 12]. The PGR is attached to the waist of the patient, and records the above signals at a sampling rate of 10 ms. The data are automatically stored in a micro SD card. When recording is completed, the absolute value of acceleration vectors (*a*; *a* ^2^ = *a*_*x*_^2^ + *a*_*y*_^2^ + *a*_*z*_^2^) is calculated and graphically displayed on the PC. A fully charged PGR can achieve 40 hours of continuous recording.

#### 2.2.2. Identification of Acceleration Induced by Gait Motion

The acceleration vectors caused by stepping can be distinguished from those by other limb and trunk movements or by unexpected artifacts, based on the mathematical method of  “pattern matching,” as reported previously [11, 12]. 

The acceleration vectors related to stepping can be distinguished from those related to other movements, since the former has steep curves and appears rhythmically. Furthermore, based on the mathematical method of  “pattern matching,” we identified the acceleration vectors caused by stepping from those by other trunk and limb movements. First, attention was focused on relatively strong signal region (e.g., *a* > 1 m/s^2^) in the acceleration time series, and a three-dimensional template wave (*a*_*x*_, *a*_*y*_, *a*_*z*_) with a duration of about 0.5 sec was arbitrarily chosen around a local maximum point from that region (Figure 1(b)). Then, the cross-correlation CC(*t*) was calculated between this wave and the whole time series at each time *t* using the following formula:

(1) $$\begin{array}{c} \text{CC}\left(t\right)\frac{\left(1/p\right)\sum_{i=1}^{p}\left[a_{x}\left(i\right)a_{x}\left(i+t\right)+a_{y}\left(i\right)a_{y}\left(i+t\right)+a_{z}\left(i\right)a_{z}\left(i+t\right)\right]}{{\left\{(1/p)\sum_{i=1}^{p}\left[a_{x}{\left(i\right)}^{2}+a_{y}{\left(i\right)}^{2}+a_{z}{\left(i\right)}^{2}\right]\right\}}^{1/2}{\left\{(1/p)\sum_{i=1}^{p}\left[a_{x}{\left(i+t\right)}^{2}+a_{y}{\left(i+t\right)}^{2}+a_{z}{\left(i+t\right)}^{2}\right]\right\}}^{1/2}}, \end{array}$$

where *p* is the length of the template wave. The obtained cross-correlation is a scalar time series, showing pronounced rhythmic peaks even when the initial signal is too noisy to visualize periodicity with naked eyes. If the acceleration change is caused by gait motion, the CC(*t*) peaks exhibit alternate changes in magnitude with time due to left/right body sway during walking. Thus, we can pick up correct peaks corresponding to one gait cycle (Figure 1(c)). This “pattern matching method” also enabled us to distinguish the acceleration vectors caused by stepping from unexpected and large artifacts. Moreover, although the PGR moved from the original position during the recording and the form of the acceleration vectors changed, we were able to identify the acceleration vectors continuously. 

#### 2.2.3. Long-Term Monitoring of the Gait Cycle and Acceleration

Changes in gait cadence (steps per minute) and gait accelerations cycle were examined during the 24 hrs. The cadence and acceleration represent the mean values recorded every hour. Data were excluded when the step rate was <20/hr.

#### 2.2.4. Estimation of “Gait Acceleration-Cycle Plot” and “% Alteration Range”

**Figure 2:**
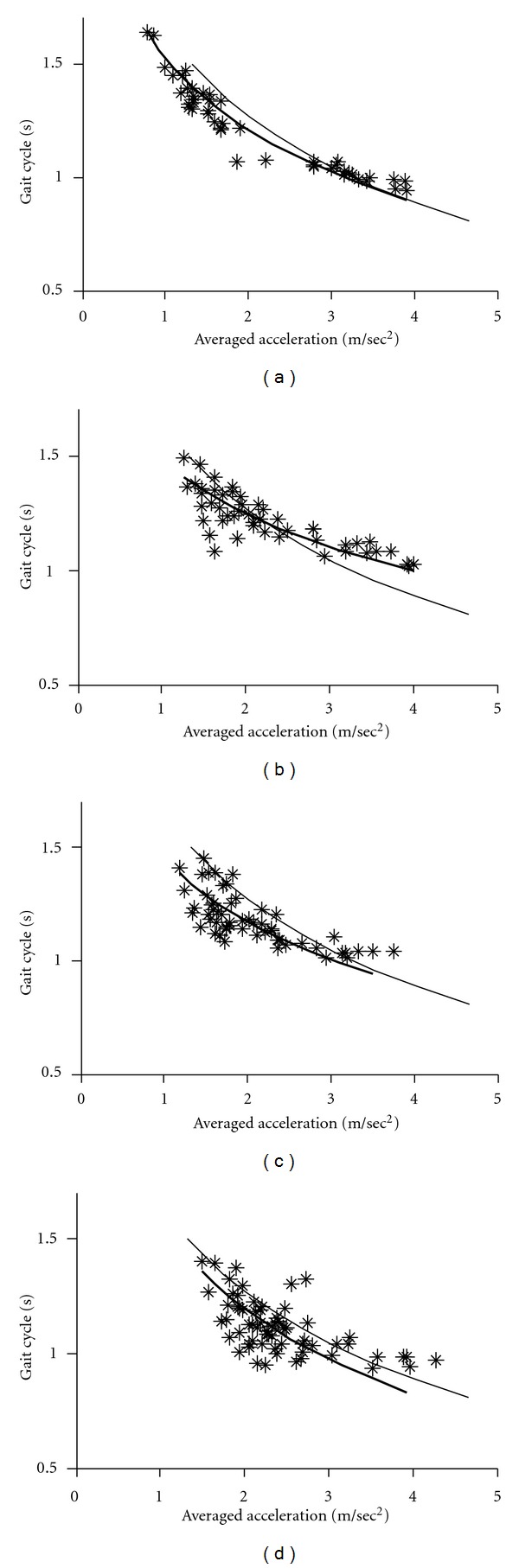
Examples of *normal pattern* of the *gait acceleration-cycle plot. *The percentage of change range was >75% for both the gait acceleration and cycle. (a) Patient number 77, (b) Patient number 83, (c) Patient number 72, and (d) Patient number 26.

**Figure 3:**
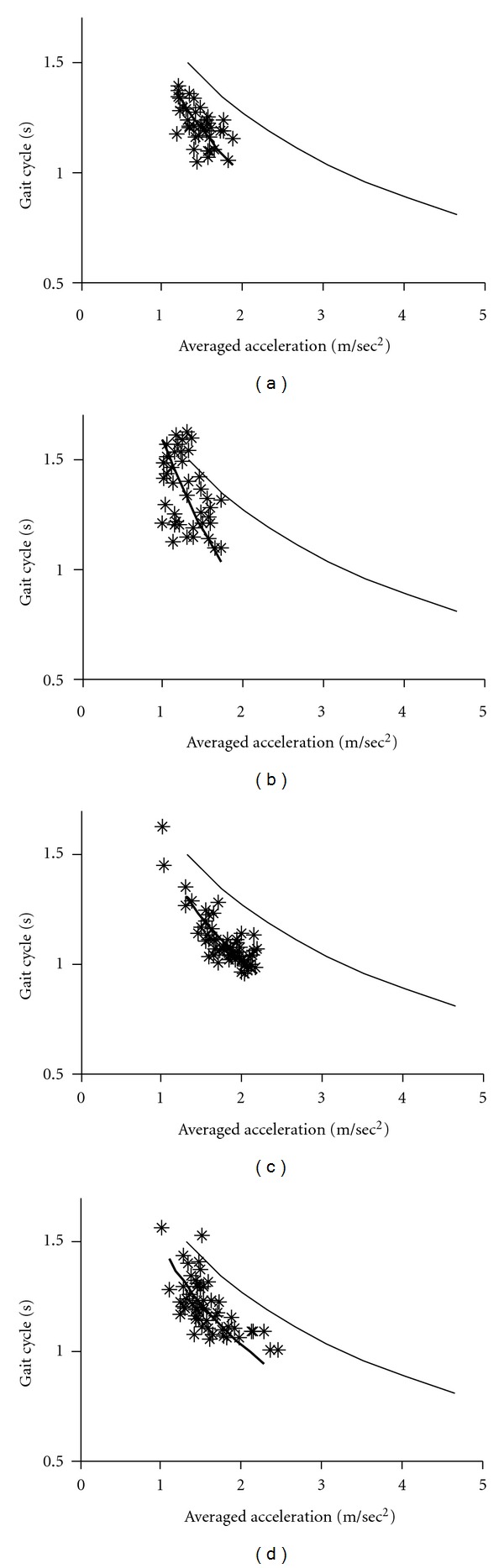
Examples of the *steep pattern* of the *gait acceleration-cycle plot.* The percentage of change range of gait acceleration was 34% in (a) (patient number 11), 38% in (b) (patient number 3), 42% in (c) (patient number 58), and in 45% (d) (patient number 79).

**Figure 4:**
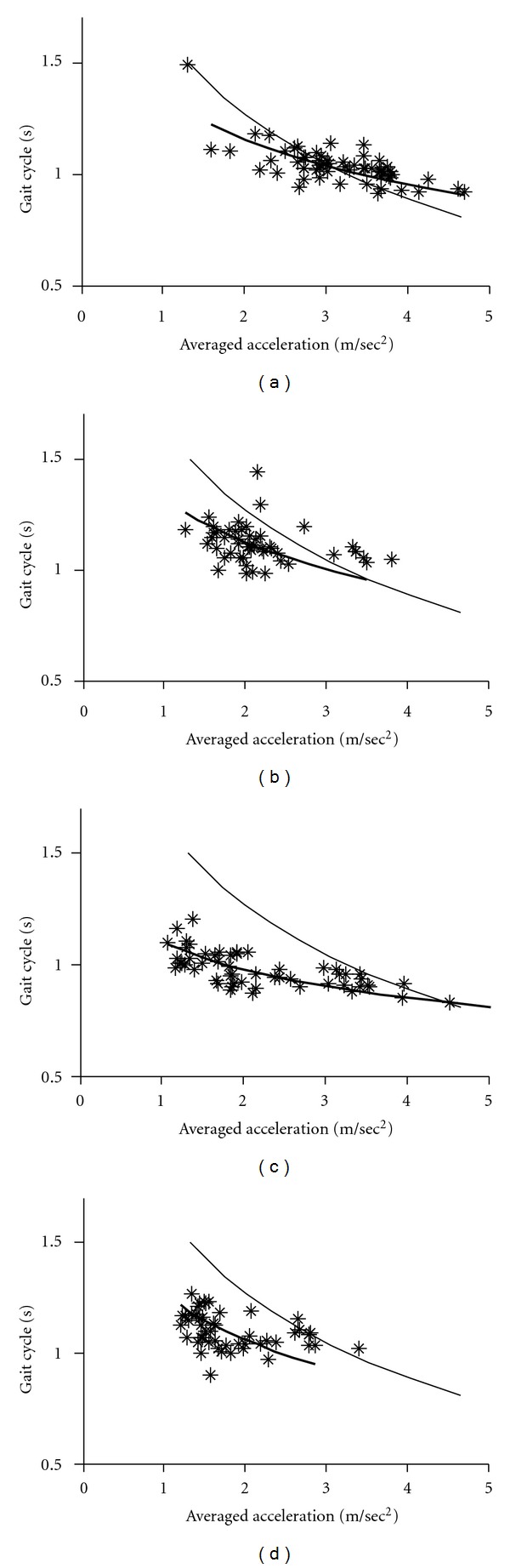
Examples of the *flat pattern* of the *gait acceleration-cycle plot. *The percentage of change range of the gait cycle was 57% in (a) (patient number 16), 63% in (b) (patient number 5), 67% in (c) (patient number 4), and 72% in (d) (patient number 44).

Since gait acceleration correlates with the floor reaction forces, we measured gait acceleration as an index of floor reaction forces. The gait-related accelerations and duration of gait cycles were averaged for each 10 min recording (see Figures 2–4). The graphs shown in these Figures indicate that changes in the gait acceleration correlate with the gait cycle duration. The averaged logarithmic values showed normal distribution and the slope of the regression line was calculated using principal component analysis. Figures 2–4 show the *gait acceleration-cycle plot *of each PD patient with the corresponding regression line (thick line). The regression line, obtained from the summed average of 17 normal controls, is also shown (thin line).

In the *gait acceleration-step cycle plot*, which was obtained from one trial in each patient, the mean and standard deviation were calculated for gait acceleration and cycle. Here, the SD was determined for an index of the alteration range. The interindividual mean of SD was calculated for the 17 normal controls. Thus, the ratio of the SD for each patient to the interindividual mean of SD for the normal controls was defined as “*% alteration range.*”

In analysis of the *percentage of alteration range*, we used a cut-off level as 75%. For gait acceleration, the SD of the controls was 0.55 (m/sec^2^) and, therefore, the 75% cut-off level was 0.41 (m/sec^2^). For the gait cycle, the SD of the controls was 0.11 (sec) with 0.082 (sec) representing the 75% cut-off level.

## 3. Results

The mean gait acceleration in PD was 2.00 ± 0.38 (m/sec^2^) (*n* = 40) and was significantly lower than that of the normal controls (2.78 ± 0.42 (m/sec^2^), *n* = 17) (*P* < 0.001). The *percentage of alteration range* was less than 75% in 16 of 40 patients. On the other hand, the mean gait cycle in PD patients was 1.14 ± 0.10 (sec) (*n* = 40), which was not significantly different from that of the normal controls (1.12 ± 0.11 (sec), *n* = 17). The percentage of* alteration range* was less than 75% in 12 of the 40 patients.

**Figure 5:**
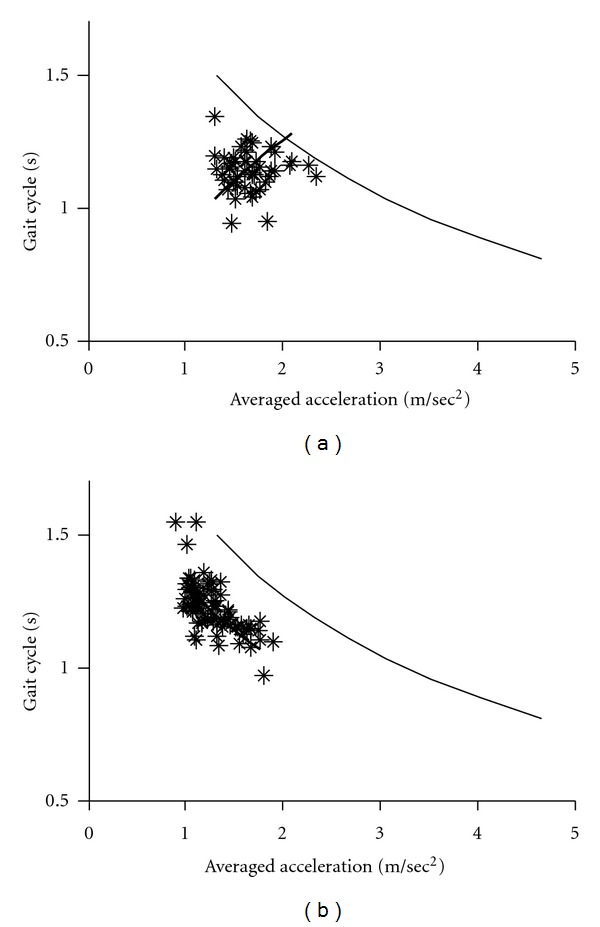
Examples of the *lump pattern* of the *gait acceleration-cycle plot. *The percentage change range was <75% both for gait acceleration and cycle. (a) patient number 1, (b) patient number 103.

Figures 2–5 show the *gait acceleration-cycle plots *based on percentage of* alteration range* of the gait acceleration and cycle. The regression curves of patients with percentage of alteration range >75% were similar to those of the controls (*n* = 16) (Figure 2). However, when percentage of* alteration range* of the acceleration or cycle decreased, the regression curve deviated showing the following three patterns.  Figure 3 shows the *gait acceleration-cycle plots* of patients with percentage of* alteration range* of the gait acceleration of <75%. Due to the narrow distribution of the gait acceleration values, the slope of the regression curve was steep (*steep type*). This pattern was noted in 12 patients.  Figure 4 shows the *gait acceleration-cycle plots* of patients with percentage of* alteration range* of the gait cycle of <75%. Due to the narrow distribution of the gait cycle values, the slope of the regression curve was flat (*flat type*). This pattern was noted in 8 patients.  Figure 5 shows the *gait acceleration-cycle plots* of patients with percentage of* alteration range* of <75% of both the gait acceleration and cycle. Interestingly, the data of these patients showed a narrow scatter (the *lump type*). The *lump pattern* was noted in 4 patients. Due to a narrow scatter, the slope of the regression curve became reverse in one patient (see Figure 5(a)).

There were no significant differences in UPDRS III between patients with the *normal type* (19.1 ± 8.0) and those with *steep, flat,* and *lump patterns* (19.3 ± 6.7) (*P* = 0.93). 

## 4. Discussion

The present results using our newly developed long-term monitor device demonstrated narrowness in changes in gait accelerations and gait cycles. The* gait acceleration-cycle plots*, obtained from 24 hrs records of daily walking, showed deviation from the normal curves, and the extent of deviation was dependent on the decrease in the percentage of* alteration range*. The percentage of* alteration range* represented the ratio of the SD for each patient to the inter-individual mean of SD for the normal controls

One possible reason for the narrow range of acceleration and cycle is the lack of movements within the house. With worsening of akinesia, physical activity tends to decrease in these patients, with subsequent decrease in walking even inside the house [11, 12]. However, the patients of this study were asked to come to our hospital for wearing on and off the device during the recordings. Thus, such travel to and from the hospital ensured some degree of walking.

An alternative explanation for the narrow alteration range is related to a deficit in scaling of motor parameters, an elementary symptom in parkinsonism. Flowers [13] postulated that PD patients can only execute movements of different amplitudes at a single and slow velocity and cannot increase their movement velocity. Consistent with this notion, the peak EMG activity that can be generated in a muscle burst is limited in PD patients [14]. The present finding might similarly suggest that PD patients could not vary the force and rhythm in their daily routine walking, and that PD patients consequently walk with a narrow range of floor reaction forces and step cycles. We reported previously the narrowness of floor reaction forces by calculating the slope of the regression line of the *gait acceleration-cycle plot* [11]. In the present study, we confirmed this change using another method that measures the range of gait accelerations. The results confirmed a restricted range of change in the gait cycle. Although the mean values of the cycle was unchanged compared with controls, the range of the mean ± 1 SD range was narrow.

The *gait acceleration-step cycle plot *showed that when a normal subject walks with a strong floor reaction force, the cycle increases simultaneously. The characteristic change in the shape of this curve is the narrowness of the range of force and/or rhythm. In patients who walked with a narrow range of change in gait acceleration (i.e., floor reaction forces), the slope of the regression curve was steep (*steep type*). This type reflects short-step walking pattern, in which no effective floor reaction force is achieved despite the fast step [16]. In contrast, patients who walked with a narrow range of change in the gait cycle, the regression curve was flat (*flat type*). Under this condition, the patient always walks with the preferred cycle, that is, monotone walking. Other patients walked with a narrow range of both acceleration and cycle, with small scatter of gait acceleration and cycle data (*lump type*).

Assessment of daily activities during 24 hrs should take into consideration the contamination with involuntary movements. Resting tremor, occurring periodically at 5-6 Hz, would be easily excluded using our analysis algorithm. However, dyskinesia is difficult to identify, since it occurs irregularly and in a variety of forms. Severe disturbances of the gait parameters, associated with high activities, could reflect dyskinesia.

Taken together, our new device, which allows long-term monitoring with simultaneous recording of gait acceleration and cycle, identified deficits in the scaling of gait parameters, a potential pathomechanism underlying PD. Analysis of daily physical activities, including walking, using this device might extend our understanding of motor symptoms of PD.

## 5. Conclusion

To examine the range of gait acceleration and cycle in daily walking of patients with Parkinson's disease (PD), we compared the gait of 40 patients with PD and 17 normal controls by using a newly developed long-term monitoring device that extracts gait-related accelerations from overall movements-related accelerations.The range of change in gait acceleration, relative to the control, was less than 75% in 12 patients. The range of change in gait cycle was less than 75% in 8 patients. The range of changes in both parameters was less than 75% in 4 patients. The present finding of narrow changes in gait parameters supports a notion that PD patients find it difficult to shift gait parameters in response to varying situations during walking.

## Figures and Tables

**Table 1:** Profile of patients, divided according to the percentage of alteration range of gait acceleration.

Patient Number	Sex	Age (yrs)	Hoehn and Yahr stage	UPDRS III	Duration of illness (yrs)	Subjective off	Percentage of alteration range in	
							Acceleration	Cycle
Normal pattern								

77	M	71	2	9	0.7		176	200
99	M	59	3	27	0.9		176	155
83	M	54	1	6	1.0		143	109
46	M	68	3	30	4	off	125	83
71	M	61	1	13	2.1		120	118
97	M	74	3	25	9.0	off	120	109
42	F	64	3	21	13	off	116	100
72	M	66	2	14	0.8		105	100
26	F	73	1	11	0.5	off	96	100
73	M	63	2	22	2.2		94	118
31	M	75	3	28	12		94	127
69	F	68	3	24	4	off	94	84
32	F	73	1	10	10		82	86
25	M	72	3	29	3.5	off	80	109
27	F	63	2	23	0.5		78	76
75	M	60	1	13	4.1		78	109

Steep pattern								

47	M	65	1	10	1.5	off	75	100
40	M	71	3	24	9	off	73	87
23	F	56	3	18	3	off	70	100
28	F	70	2	30	5	off	71	118
94	F	75	3	16	11.8	off	65	173
43	F	70	1	9	3		60	76
78	M	68	2	19	4.8	off	60	87
38	F	65	3	16	3		51	88
79	F	65	3	18	3.9		45	91
58	M	65	3	13	2		42	100
3	M	63	1	16	6		38	146
11	M	59	3	15	8	off	34	82

Flat pattern								

4	M	63	3	29	5	off	176	67
16	M	54	3	23	3	off	116	57
19	M	65	3	26	1.5	off	109	74
5	M	72	2	31	2	off	98	63
96	F	68	3	17	2.1		96	65
44	M	73	3	27	2	off	92	72
36	M	56	2	11	0.5		76	72
102	M	63	3	16	0.8		76	61

Lump pattern								

9	F	67	2	19	4.5	off	47	69
103	M	68	3	30	5.6		40	65
82	F	69	3	17	1.0		38	64
1	M	74	3	12	4		35	54
